# Liposomes Composed by Membrane Lipid Extracts from Macrophage Cell Line as a Delivery of the Trypanocidal *N*,*N*’-Squaramide 17 towards *Trypanosoma cruzi*

**DOI:** 10.3390/ma13235505

**Published:** 2020-12-02

**Authors:** Christian Rafael Quijia, Cínthia Caetano Bonatto, Luciano Paulino Silva, Milene Aparecida Andrade, Clenia Santos Azevedo, Camila Lasse Silva, Manel Vega, Jaime Martins de Santana, Izabela Marques Dourado Bastos, Marcella Lemos Brettas Carneiro

**Affiliations:** 1 Microscopy Laboratory, Department of Cell Biology, Institute of Biology, University of Brasília, UnB—Brasilia, Federal District, Brasília DF 70910-900, Brazil; christianqui47@gmail.com; 2 Laboratory of Nanobiotechnology, Embrapa Genetic Resources and Biotechnology, Parque Estação Biológica, PqEB, Av. W5 Norte (Final) Caixa Postal 02372, Brasília DF 70.770-917, Brazil; cinthiabonatto@gmail.com (C.C.B.); luciano.paulino@embrapa.br (L.P.S.); 3 Pathogen-Host Interface Laboratory, Department of Cell Biology, Institute of Biology, University of Brasília, UnB—Brasilia, Federal District, Brasília DF 70910-900, Brazil; andrademilene@unb.br (M.A.A.); clenia.azevedo@gmail.com (C.S.A.); camila.lasse@gmail.com (C.L.S.); jsantana@unb.br (J.M.d.S.); 4 Department of Chemistry, University of the Balearic Islands, Palma on the Island of Majorca, Carretera de Valldemossa, km 7.5, 07122 Palma, Illes Balears, Spain; manel.vega@uib.es

**Keywords:** Chagas, trypanocidal effect, cytotoxicity, nanostructures, mimetic lipid membrane

## Abstract

Chagas is a neglected tropical disease caused by *Trypanosoma cruzi*, and affects about 25 million people worldwide. *N*, *N*’-Squaramide 17 (S) is a trypanocidal compound with relevant in vivo effectiveness. Here, we produced, characterized, and evaluated cytotoxic and trypanocidal effects of macrophage-mimetic liposomes from lipids extracted of RAW 264.7 cells to release S. As results, the average hydrodynamic diameter and Zeta potential of mimetic lipid membranes containing S (MLS) was 196.5 ± 11 nm and −61.43 ± 2.3 mV, respectively. Drug entrapment efficiency was 73.35% ± 2.05%. After a 72 h treatment, MLS was observed to be active against epimastigotes in vitro (IC_50_ = 15.85 ± 4.82 μM) and intracellular amastigotes (IC_50_ = 24.92 ± 4.80 μM). Also, it induced low cytotoxicity with CC_50_ of 1199.50 ± 1.22 μM towards VERO cells and of 1973.97 ± 5.98 μM in RAW 264.7. MLS also induced fissures in parasite membrane with a diameter of approximately 200 nm in epimastigotes. MLS showed low cytotoxicity in mammalian cells and high trypanocidal activity revealing this nanostructure a promising candidate for the development of Chagas disease treatment.

## 1. Introduction

Chagas disease or American Trypanosomiasis is a neglected tropical disease considered endemic in twenty-one Latin American countries. Over recent decades, however, it has been increasingly detected in the United States of America, Canada, and many European countries. This is due, mainly, to population mobility between Latin America and the rest of the world and has caused more than 7000 deaths per year and over 25 million people at risk of infection [1]. It is transmitted to humans mainly through the feces of triatomine insects (vector) with hematophagous habits [2].

**Figure 1:**
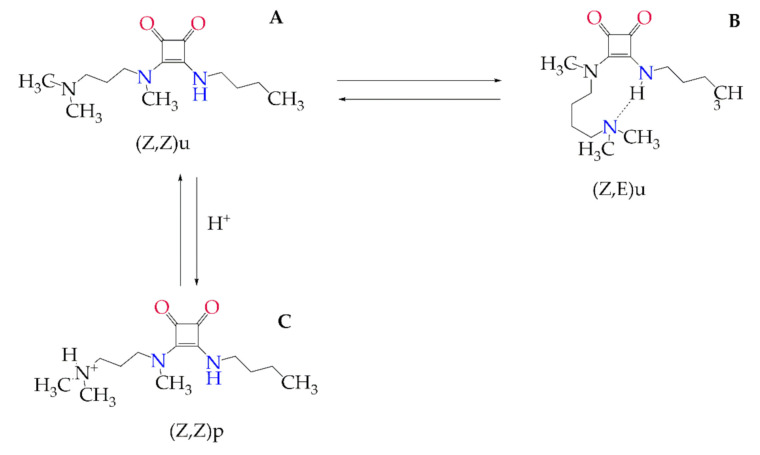
(**A**) Chemical structure of *N, N’-*Squaramide 17 (S); (**B**) Conformational transitions between (*Z, Z*) and (*Z, E*) Rotamers of Aminosquramide 17 (the subscripts “ *u*” and “ *p* ” indicate neutral and protonated forms, respectively); (**C**) Conformational transitions between (*Z, Z*) and (*Z, E*) Rotamers of Aminosquramide 17 (the subscripts “u” and “p” indicate neutral and protonated forms, respectively) [4].

Currently, chemotherapeutics are based on nitro heterocyclic compounds, such as nitrofuran (nifurtimox) and nitroimidazole (benznidazole). However, these therapeutic options are unsatisfactory, given their limited effectiveness in preventing the chronic stage of the disease [3]. As a neglected disease, investigations focusing on drug discovery are insufficient, revealing the urgency search for active molecules and new formulations with therapeutic potential. A promising treatment for this disease with low-cost synthesis is the therapeutic agent *N, N’*-Squaramide 17 (S) (Figure 1).

S comply with Lipinski’s rule of five (Ro5) as they are low molecular mass (267.4 Da); only one hydrogen bond donor; partition coefficient log P = 0.59; polar surface area values <100 Å^2^; the pKa is 8.8 in aqueous solution, therefore, at physiological pH, both the acidic and the basic forms coexist in solution. A previous study with S showed a 67% reduction of parasitemia in the acute phase of infection in a mice model after 40 days of treatment compared to the control [4]. The potential use of this molecule in the development of new drugs was, therefore, suggested. It is known that bioactivity of drugs can be enhanced using nanobiotechnology, due to inherent properties of nanostructured systems such as (1) prolonged circulation, (2) sustained release of the drug, and (3) greater specificity at the target site [5]. Nanostructures can be designed to obtain specific physicochemical properties such as size, shape, surface charge, and hydrophobicity/hydrophilicity, and thus achieve research goals [6]. 

There are a limited number of studies using nanotechnology seeking the treatment of Chagas disease. One barrier for the effective treatment of Chagas disease may be the fact that the parasite easily spreads throughout the body, infecting several cell types, and evades the immune system [7]. Liposomes are nanosystems with potential applications as active vectors of drug delivery due to their ability to improve drug’s pharmacological action by their greater solubility, stability, biodistribution, and their mode of release [8]. A rational strategy that has arisen recently is the use of liposomes extracted from cell membranes (mimetic lipid membranes) as carriers of drugs, due to their biocompatibility, cell uptake, and expected low immunogenicity [9,10,11,12,13]. 

Here, we present the preparation and characterization of mimetic lipid membranes based on lipids extracted from macrophage cells (RAW 264.7 strain) as a strategy to controlled release of the trypanocidal agent S. The nanostructures were characterized by several approaches such as hydrodynamic diameter, polydispersity index (PdI), Zeta potential, stability on the first and tenth day after synthesis (dynamic ligth scattering and electrophoretic mobility), size (dry diameter), shape (transmission electron microscopy—TEM and atomic force microscopy—AFM), chemical behavior (infrared vibration spectroscopy—IR), and entrapment efficiency (UV-Vis spectrophotometry). Finally, the nanostructures’ toxicity on mammalian cells and their trypanocidal activity (CL-Brener) were also evaluated, as well as the damage effects on the parasites using scanning electron microscopy (SEM).

## 2. Materials and Methods 

### 2.1. Cells and Parasites

VERO (ATCC CCL81) and RAW 264.7 cells (ATCC TIB71) were maintained in RPMI-1640 supplemented with 10% fetal bovine serum (FBS) (Sigma Aldrich, St. Louis, MO, USA) without phenol red at 37 °C and 5% CO_2_ [14]. *T. cruzi* (strain CL-Brener) epimastigote form was maintained at 27 °C in axenic culture RPMI-1640 containing 10 U/mL penicillin, 25 μg/mL streptomycin, 25 mM HEPES buffer, 0.03 M Hemin, and 200 mM glutamine supplemented with 10% inactivated FBS [15]. Trypomastigotes were obtained from monolayers of infected VERO cells grown at 37 °C in an atmosphere with 5% CO_2_ in RPMI-1640, pH 7.4, supplemented with 10% FBS and 100 μg/mL gentamicin. 

### 2.2. Nanostructure Synthesis

#### 2.2.1. Lipid Extraction from Macrophages RAW 264.7

For lipids extraction from RAW 264.7 macrophage cells, about 1 × 10^8^ cells/mL were centrifuged and washed in phosphate-buffered saline (PBS) and resuspended in deionized water for lysis. Then, cells were centrifuged and placed in deionized water-hypotonic solution to finally obtain the cell pellet and were stored at −40 °C until use. The lipids extraction was performed according to Bonatto et al. [16] with some adaptations. Briefly, after cells had been resuspended in 400 µL of ultrapure water, forming a homogeneous solution, solvents were sequentially added followed by vortex for 5 min: 1250 µL of chloroform, 2400 µL of methanol, 1250 µL of chloroform, and 1250 µL of water. Next, samples were centrifuged at 3.040× *g* for 5 min for organic phase separation containing the lipids. This phase was collected and subjected to the rotary evaporator for 1 h in a water bath (Quimis, São Paulo, Brazil) at 40 °C and pressure of 200 Pa, for chloroform evaporation and consequently obtaining the formation of the lipid film.

#### 2.2.2. Production of Nanostructured Systems by Extrusion Method

After obtaining the phospholipid films, 5 mL of ultrapure water were added to produce the following compositions: (1) Empty mimetic lipid membranes (MLV) and (2) Mimetic lipid membranes containing *N, N’-*Squaramide 17 (MLS) at 0.032 M. The solutions were homogenized for 10 min and then submitted to the extrusion process with syringes (15 repetitions were carried out) through polycarbonate membranes with 100 nm pores (Avanti Polar Lipids). 

### 2.3. Entrapment Efficiency

The MLS nanostructures were evaluated in terms of drug entrapment efficiency (EE), established as a ratio between the encapsulation (ED) over the initial quantity of the drug (ID), according to the Equation (1) below:

(1) $$EE\;\left(\%\right)=\frac{ED\;\left(mg\right)}{ID\;\left(mg\right)}\times 100$$

For the determination of EE, the S-loaded nanostructures were filtrated in 100 kDa Amicon filters (Amicon^®^ Ultra 0.5 mL centrifuge filters - Millipore, Darmstadt, Germany) at 300× *g* for 10 min, 4 °C [17]. Unentrapped drug (filtered content) was collected and the absorbance was measured at 320 nm [18] using a biophotometer (Eppendorf BioPhotometer^®^ 6131, Hamburg, Germany) (Figure S1). S concentration was calculated from a previously established absorbance calibration curve (R^2^ = 0.98) and MLV absorbance was also performed to determine the background in this sample. Experiments were performed in triplicates. 

### 2.4. Nanostructure Characterization

#### 2.4.1. Infrared Vibrational Spectroscopy (IR) Analysis

Mimetic membranes were lyophilized using sucrose (350 mM) as a cryoprotectant at a ratio of 1:1, 1 mL of the nanostructures (MLV, MLS), and 1 mL of sucrose [19]. Spectra (4000–400 cm^−1^) were submitted to infrared vibrational spectroscopy (IR) analysis in a Perkin-Elmer model 400 IR spectrometer (Perkin-Elmer Inc., Boston, MA, USA). The samples were mixed with potassium bromide (KBr) in an agate mortar and then introduced into pellets for reading, with a resolution of 2 cm^−1^, via analysis of 32 scans. Data were processed using the software OriginPro version 2018 (OriginLab Corporation, Northampton, MA, USA) [20].

#### 2.4.2. Dynamic Light Scattering and Electrophoretic Mobility for Hydrodynamic Diameter and Zeta Potential Evaluation

Hydrodynamic diameter of the formed nanostructures was determined by dynamic light scattering (DLS) in a ZetaSizer Nano ZS (Malvern Instruments Ltd., Malvern, UK) using He-Ne laser (4 mW) at 633 nm. Three measurements were performed at 25 °C under pH 7.0 and light scattering measured at an angle of 173° in automatic mode. Additionally, electrophoretic mobility measurements were obtained for the assessment of the Zeta potential in manual mode with 20 readings. Particles size (average hydrodynamic diameter), polydispersity index (PdI), as well as the Zeta potential, were acquired and processed using ZetaSizer software (Malvern Instruments 7.1, Malvern, UK) [21]. The stability of the nanostructures from the first and tenth day after synthesis and storing at 4 °C was also analyzed.

#### 2.4.3. Atomic Force Microscopy (AFM) Analysis 

AFM analysis was performed on dynamic mode using a SPM-9600 equipment (Shimadzu, Corporation, Kyoto, Japan) with rectangular cantilevers integrated with conical tips, spring constant and a resonance frequency of about 42 N/m and 300 kHz, respectively. For the analysis, 1 µL of MLS and MLV samples were diluted 100× in ultrapure water, deposited onto freshly-cleaved mica surfaces, and air-dried in a protected environment. Analyses were performed at ∼21 °C. Up to ten images of each sample were acquired in areas of 25 µm^2^ (5 µm × 5 µm) with a resolution of 512 × 512 lines. Images were processed for XY plane correction using the software Gwyddion 2.56 verion 2018 (Gwyddion, Okružní, Czech Republic). 

#### 2.4.4. Transmission Electron Microscopy (TEM) Analysis

For TEM analysis (JEM-1011) (JEOL, Tokyo, Japan), samples (MLS and MLV) were diluted at a ratio of 1:1000 in deionized water and deposited onto Formvar-coated copper grids (200 mesh). Samples were then contrasted with 1% osmium tetroxide (OsO_4_) for 7 min. Therefore, 10 images were acquired using an 80 kV beam voltage, and the modal dry diameter of 100 nanostructures was measured through the Image-Pro Plus software version 1.8.0 (Media Cybernetics, Silver Spring, MD, USA) and nanostructures were evaluated according to the distribution classes [22].

### 2.5. Trypanocidal Activity and Cytotoxicity

#### 2.5.1. Cytotoxicity Assays in VERO and Macrophage RAW 264.7 Cells

The reference drug benznidazole (BZ) was dissolved in dimethyl sulfoxide (DMSO) at 5% (*w/v*) at 0.002 M (stock) and the final concentration of DMSO in the experiments never exceeded 0.25% (*v/v*) to not affect the cell viability [22]. The MLV, MLS, and free S were dispersed or dissolved in deionized water. The final concentration of these treatments in the experiments never exceeded 0.01% (*v/v*). VERO cell line and RAW 264.7 macrophages were cultured in RPMI medium supplemented with 10% FBS at a density of 1 × 10^4^ cells/wells in 96-well microplates for 48 h. After this period, cells were treated with BZ, S, MLV, or MLS serially diluted at the dosages of 800 to 6.25 μM (modified from OLMO et al. [4]) over a period of 72 h. Thereafter, 20 μL of resazurin—(0.39 mM, Sigma Aldrich, St. Louis, MO, USA) was added to each well, followed by incubation for 2 h, and then fluorescence was read on a microplate reader SpectraMax M5 (570 nm_ex_/595 nm_em_) (Molecular Devices, Sunnyvale, CA, USA) [23]. Percentage of cell viability was calculated using the equation below, where A is the arbitrary fluorescence units (AFU) of the treatments, B is AFU culture medium with resazurin and C is AFU of the control group.

(2) $$\%\;Viability=\frac{A-C}{C-B}\times 100$$

The adjusted equations were used to calculate the concentration needed to kill 50% of VERO cells and macrophage RAW 264.7 cells (CC_50_) by nonlinear regression in GraphPad Prism version 7.0 (GraphPad Software, San Diego, CA, USA).

#### 2.5.2. Trypanocidal Activity of Nanostructures

Treatments with BZ, S, MLV, and MLS were performed using serial dilution at 100 to 6.25 μM [4] and were prepared in a 96-well plate. Then, 2.5 × 10^5^ epimastigotes/mL were resuspended in 150 μL and added to the microplates. After 48 h, resazurin was added (20 μL/well of resazurin −3 mM), incubated for further 24 h, and fluorescence was recorded as described above [24]. Nanostructures’ effect on amastigotes was accessed in VERO cell line grown in RPMI medium containing 10% FBS and seeded at a density of 1 × 10^4^ cells/well in 24-well microplates. After 48 h, cells were infected with trypomastigotes at a ratio of 10:1 during 24 h, followed by removal of reminiscent extracellular parasites by washing with PBS. Then, cells were cultivated in the presence of BZ, S, MLV, and MLS added to the culture medium in a serial dilution of 100 to 6.25 μM for 72 h [4]. Cells were fixed and stained with Kit for Fast Staining in Haematology (Figure S2), and the number of amastigotes was determined by counting 200 cells for each experimental group distributed in random microscopic fields. The IC_50_ values were determined by nonlinear regression in GraphPad Prism version 7.0 (GraphPad Software, San Diego, CA, USA). Selectivity indices (SI) were calculated using the formula: SI = CC_50_ cytotoxicity in VERO cells/IC_50_ cytotoxicity in intracellular amastigotes.

#### 2.5.3. Ultrastructural Changes in Epimastigotes 

Epimastigotes of *T. cruzi* were grown at a density of 5 × 10^5^ cells/mL in RPMI culture medium containing the compounds tested at concentrations of 7.91 µM (BZ), 6.56 µM (S), and 7.93 µM (MLS) corresponding to the half of IC_50_ of each compound. After 72 h these cultures were centrifuged at 1600× *g* for 10 min. Parasites were washed in PBS and fixed for 1 h at room temperature with 2% formaldehyde and 2.5% glutaraldehyde in sodium cacodylate buffer (0.1 M, pH 7.2). Samples were then washed in 0.1 M cacodylate buffer and adhered to coverslips previously coated with poly-L-lysine (Sigma Aldrich, St. Louis, MO, USA). After adhesion, post-fixation was performed using a solution of 1% OsO_4_ containing 0.8% potassium ferrocyanide (K_4_Fe (CN)_6_). Then, parasites were dehydrated in increasing concentrations of acetone (30%, 50%, 70%, 90%, and 100%), subject to critical-point drying, metalized with gold, and observed in a scanning electron microscope (SEM)- (JEOL JSM-7000F, Tokyo, Japan). Sample images were visualized using a 15 kV beam voltage.

### 2.6. Statistical Analysis

The results were expressed as mean (± standard deviation of the mean) of three independent experiments, and one-way or two-way analysis of variance (ANOVA) were performed respectively, followed by the post- Dunnett’s test (two-tailed) comparing the treatments BZ, S, and MLV with MLS. Statistically significant differences were considered when *p* < 0.05. All analyses were performed in GraphPad Prism version 7.0 (GraphPad Software, San Diego, CA, USA).

## 3. Results and Discussion

### 3.1. Characterization of Nanostructured Systems 

**Figure 2:**
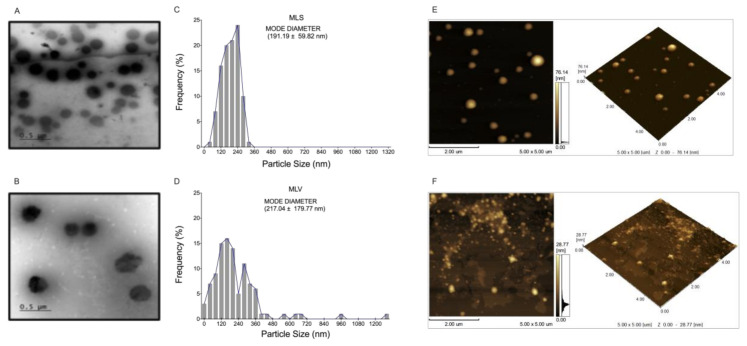
Characteristics of nanostructures: 1.- Morphological analyzed by transmission electron microscopy (TEM): (**A**) Mimetic lipid membranes containing *N, N’*-Squaramide 17 (MLS) and (**B**) Empty mimetic lipid membranes (MLV). 2.- Class distribution histograms (*n* = 100 particles) referring to the particle size values evaluated by transmission electron microscopy (TEM): (**C**) Mimetic lipid membranes containing *N, N’*-Squaramide 17 (MLS) and (**D**) Empty mimetic lipid membranes (MLV). 3.- Characteristics of nanostructures analyzed by atomic force microscopy (AFM): (**E**) Mimetic lipid membranes containing *N, N’*-Squaramide 17 (MLS) and (**F**) Empty mimetic lipid membranes (MLV). Acquired in dynamic mode. Scanning area 5 µm × 5 µm.

Due to its low molecular weight (267.4 Da) and its hydrophilicity [4], encapsulation of S was carried out by the passive loading method during the liposome formation process (extrusion method) [25]. Evaluation of liposome nanostructures by TEM (Figure 2) showed that the MLV membranes show an irregular shape (Figure 2B) and a modal dry diameter (Dm) of 217.04 ± 179.77 nm (Figure 2D). Otherwise, MLS presented circular vesicules and heterogeneous size (Figure 2A) with Dm of 191.19 ± 59.82 nm (Figure 2C). AFM analysis indicates that the MLS sizes were less than 200 nm in diameter (Figure 2E).

In addition to these results, MLS and MLV showed sizes (hydrodynamic dyameters) smaller than 200 nm and a PdI below 0.45 (Table 1). PdI is used to represent degree of uniformity of a particle size distribution and our results show a PDI greater than 0.4 indicating a broad polydisperse [26]. Furthermore, the Zeta potential is an important parameter that can be used to predict physical (colloidal) stability of nanostructures. A high Zeta (module value), indicates a greater stability of the system, since it could provide a repellent effect between the nanoparticles [27]. The MLV Zeta-potential was determined to −12.93 ± 1.21 mV, while that of MLS was indicative of excellent colloidal stability because of its Zeta-potential of −61.43 ± 2.30 mV (Table 1), and its structures are more colloidally dispersed (Figure 2A). In addition, MLV showed vesicles agglomeration and this may be related to the considerably lower Zeta potential (Table 1) (Figure 2F-S3B) [28,29,30,31].

### 3.2. Analysis of Nanostructured Systems by Infrared Vibrational Spectroscopy (IR) and Entrapment Efficiency

**Figure 3:**
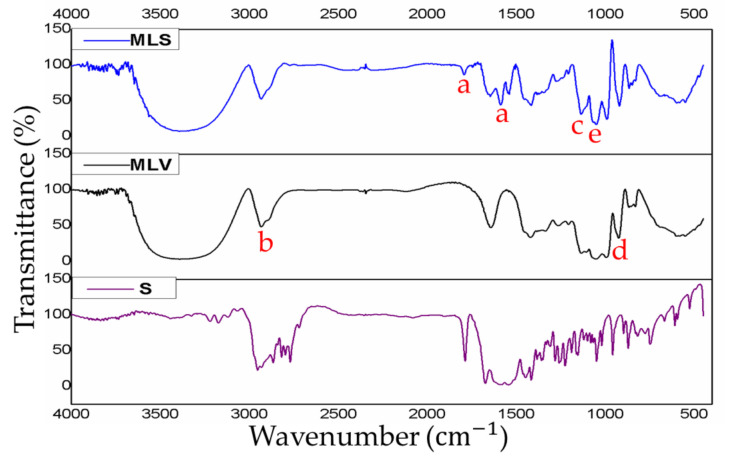
Infrared absorption spectrum of nanostructured and free drug systems analyzed by infrared (IR) vibrational spectroscopy. Upper-case letters represent compounds and nanostructures under insvestigation: S: *N, N’*-Squaramide 17, MLV: Empty mimetic lipid membranes, and MLS: Mimetic lipid membranes containing N, N’-Squaramide 17. Lower-case letters represent IR absorption peaks: **a**- 1800 cm^−1^ and 1591 cm^−1^ of C=O; **b**- 3392 cm^−1^ of R–NH_3_; **c**- 1052 c^−1^ of NH_3_^+^; **d**- 995 cm^−1^ of PO_2_^−^; **e**- 1136 cm^−1^ of POO_2_^−^.

When comparing the IR spectra of MLS and MLV (Figure 3), it is noticed a reduction in the absorption of some characteristic bands of the MLV: 3392 cm^−1^, 1052 cm^−1^, 995 cm^−1^, and 1136 cm^−1^, which are vibrations of R–NH_3_, NH_3_^+^, PO_2_^−^,and POO_2_^−^, respectively. This observation indicates that there is no detectable structural change in both nanostructures [32,33,34]. The S spectra (Figure 3), revealed different absorption peaks compared to the MLV carrier. The unique characteristics of the MLS peaks clearly showed a close association between S and MLV. The IR spectra of pure S showed absorption peaks between 1800 cm^−1^ and 1591 cm^−1^, related to the stretching vibrations C=O (carbonyls) [35,36]. These same sections were also observed in the MLS sample that was analyzed, thus confirming the interaction of the S and the MLV. In addition, the results of the entrapping efficiency of S was estimated at 73.35% ± 2.05% by biophotometry at 320 nm (Table S1). 

### 3.3. Evaluation of the Stability of Nanostructures 

**Figure 4:**
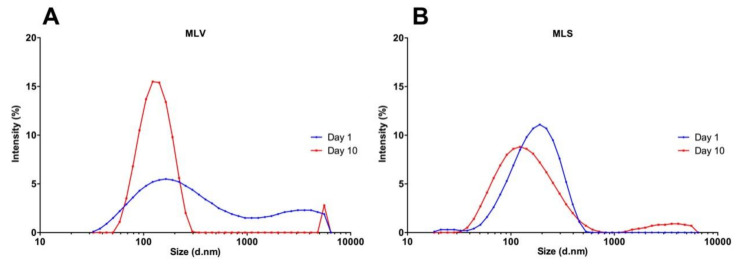
Analysis particle size distribution measured by dynamic light scattering (DLS). Distribution of the MLS (**A**) and MLV (**B**). The first day (blue line) and tenth day (red line).

Previous studies have shown that small vesicles can interact with each other and form larger liposomes during storage, showing agglomerates with sizes higher than 200 nm [37]. For this reason, an increase in MLV average hydrodynamic diameters (156.9 nm increase) was observed (Table S2). The analysis of particle size distribution of the MLS by DLS on the tenth day, showed two subpopulations of particles with approximate diameters of 140 nm and 3580 nm compared to the first day (Figure 4). This distribution is probably caused by the physical-chemical characteristics of the liposome such as the oxidation of the double bonds of the lipids and the hydrolysis of the ester bond, causing an aggregation or agglomeration of particles [38].

### 3.4. Trypanocidal Activity and Cytotoxicity 

Before starting the biological activity investigation of the compositions developed herein, MLV cytotoxicity was analyzed to verify its pharmaceutical “nanovehicle” potential and, thus, to validate the functionality of our nanostructure proposal. For this, MLV cytotoxicity was analyzed in VERO and RAW 264.7 cells employing doses from 50 μΜ to 800 μΜ, and evaluated after 72 h of treatment. No cytotoxic effects of MLV were observed on the cells analyzed, indicating that MLV composition has a potential for use as a diverse drug delivery system.

**Figure 5:**
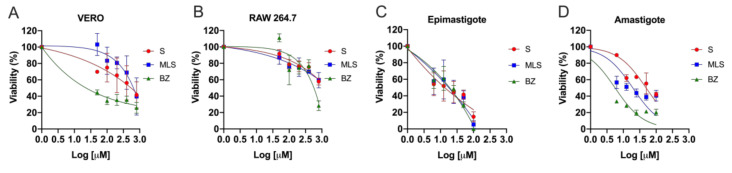
Cytotoxic and antiparasitic activity of BZ, S, MLV, and MLS. (**A**) VERO cells and (**B**) RAW 264.7 macrophages were treated with 50 μM to 800 μM of BZ, S, MLV, or MLS whereas (**C**) epimastigotes or (**D**) intracellular amastigotes were treated with 6.25 μM to 100 μM. Viability was analyzed by nonlinear regression in GraphPad Prism version 7.0 (GraphPad Software, San Diego, CA, USA).

In most studies, new compounds against *T. cruzi* are tested in epimastigote forms due to their capability to grow in axenic culture. However, as they are not found in the mammalian host, evaluations performed on these forms are only indicative of potential activity against the parasite. Therefore, a preliminary test using axenic epimastigote forms should always be complemented by a subsequent evaluation using intracellular forms (amastigotes) to assure trypanocidal activity [39]. In this study, the effect of BZ, S, MLV and MLS (6.25 μM–100 μM) was analyzed in *T. cruzi* epimastigotes and amastigotes, as well as the host cells, VERO and RAW 264.7 (50 μM–800 μM) for 72 h (Figure 5). BZ (IC_50_ = 15.81 ± 4.63 µM and 4.76 ± 4.45 µM) and MLS (IC_50_ = 15.85 ± 4.82 µM and 24.92 ± 4.80 µM) induced trypanocidal activity in both forms (epimastigotes and intracellula amastigotes) of *T. cruzi.* BZ presented the lower IC_50_ on amastigotes, MLS showed a 2-fold increase in efficacy compared with S. MLV showed no trypanocidal activity (Table 2).

When evaluating cytotoxic activity in VERO cells, CC_50_ was 736.21 μM and 1199.50 μM, after treatment with S and MLS, respectively, which is quite promising since they showed non-specific toxicity (healthy cells) and were still less cytotoxic than BZ (CC_50_ = 284.44 ± 1.25 μM). S cytotoxicity has been attributed to squaric acid, and derivatives of this compound significantly increase lipophilicity and thus have their distribution both within the cell and in lysosomes [40]. 

The SI values found for S (56.11 for epimastigotes and 14.38 for amastigotes ) and MLS (75.68 for epimastigotes and 48.14 for amastigotes) indicate a therapeutic potential according to Drugs for Neglected Diseases initiative (DNDi) [41], which preconizes that the selectivity index (SI) of a novel drug against Chagas disease must have an SI equal to or greater than 10. MLS and S showed a comparable SI for epimastigotes; however, for amastigotes, MLS was more selective than S, confirming the advantage of nanostructured system. Moreover, MLS and S associated SI values were 4 and 3 times superior to BZ regarding epimastigotes (SI = 17.99), respectively. 

There may be a correlation between the physicochemical characteristics of the particles (Table 1) and their cytotoxicity (Table 2). In this context, mammalian cells use the endocytosis process to communicate with biological environments, internalizing ions and biomolecules [42]. The drug delivery mechanism of drugs encapsulated within liposomes, suggest that these nanostructures may be adsorbed on cells surface or fused with cell membranes, thereby releasing their contents directly into the cytoplasm. Moreover, direct or mediated exchange by lipid component transfer proteins or endocytosis of liposomes that eventually accumulate in cell lysosomes may also occur [43]. 

Conversely, there is a drug internalization mechanism termed lysosomotropic–parasitotropic [44,45]. It consists of two stages, the first being the parasitophorous vacuoles formation fusing with lysosomes, creating the phagolysosomes. Also, drug-containing liposomes can be fused to the lysosomes forming the tropic lysosomes. The second step is based on the interactions and fusions between phagolysosomes and lysosomal-tropics, thus causing disintegration of the parasite. It is well known that lysosomes have an acidic and enzyme-rich environment promoting the degradation or hydrolysis of their content [46]. 

Thus, after its internalization into cells under different endocytosis processes, MLS could fuse to lysosomes and parasite-containing phagolysosomes causing its action in amastigote forms. Its trypanocidal activity is likely the result of a hydrolysis process suffered by S under different pH (2 to 8), separating it into the squaric acid and two amides (Figure 1), as previously shown by Ximenis et al. [18]. One of the advantages of this compound is that it contains a family of aromatic oligomers, which are potential hydrogen bond donors and acceptors. Thus, the squaric acid could interact with some molecules essential for the intracellular parasite metabolism causing a reduction in its replication inside the cell. Therefore, the antiparasitic activity improvement resulting from the association between liposome and S is a promising starting point in drug development to Chagas disease.

### 3.5. Structural Changes in T. Cruzi

**Figure 6:**
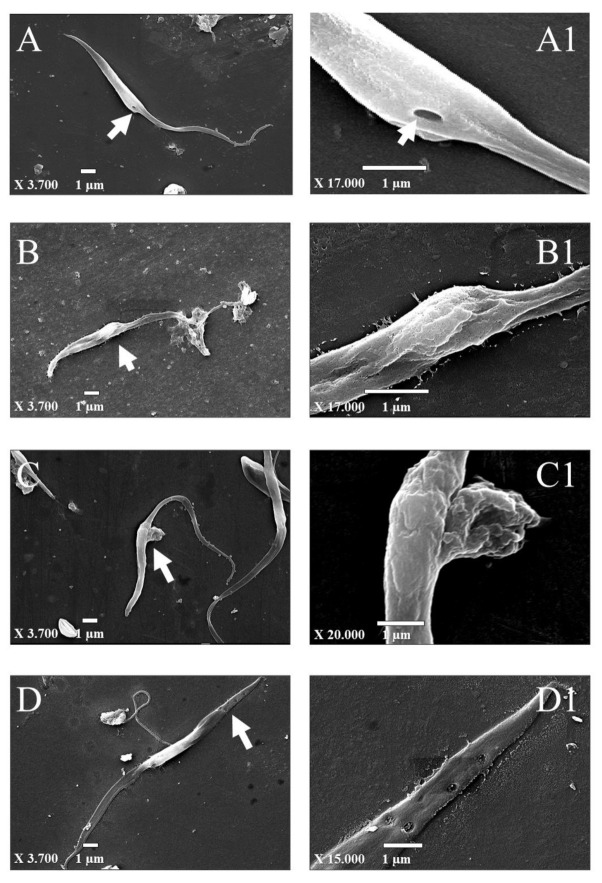
Morphological alterations in *T. cruzi* after compound treatment analyzed by SEM. Epimastigotes treated with Benznidazole at 7.91 µM (BZ); *N, N’*-Squaramide 17 at 6.56 µM (S) and Mimetic lipid membranes containing *N, N’*-Squaramide 17 at 7.93 µM (MLS) for 72 h. (**A**,**A1**) Control (without treatment); (**B**,**B1**) BZ; (**C**,**C1**) S; and (**D**,**D1**) MLS. Arrows indicate highlighted changes magnified on the right panel.

Epimastigotes were incubated for 72 h with BZ, S, and MLS, and the ultrastructural changes caused by these treatments in comparison with the control (no treatment) are shown in Figure 6. In the control (Figure 6A), it was possible to visualize specialized areas of the cell surface, such as the cytostome [47,48], which is a structure involved in the uptake of macromolecules from the medium through an endocytic process (Figure 6A1), and flagellum arising from the parasite. The cytostome–cytopharynx complex is found in the proliferative stages of the protozoan *T. cruzi* [47]. In epimastigotes (proliferative form found in the insect vector) is the major site of endocytosis [49,50], different from other protozoa from the same family (Trypanosomatidae) such as *T. brucei* and *Leishmania* sp, in which the flagellar pocket represents the only site of endocytosis and exocytosis [51].

Regarding parasites treated with BZ, cell surface alterations were observed as prominences in the cell body and distortion in the flagellum (Figure 6B,B1). Additionally, treatment with S promoted a protuberance on the cell (Figure 6C,C1), and after treatment with MLS, fissures with a diameter of approximately 200 nm in the upper cell were observed (Figure 6D,D1). In a previous study, structural alterations induced in epimastigotes by squaramide derivatives (including S), such as swollen parasites filled with electron-dense vacuoles and enlarged reservosomes were observed by TEM [4]. Pores were also described in epimastigote treated with nanoparticles of benznidazole-loaded calcium carbonate, besides the loss of membrane integrity [52]. 

## 4. Conclusions

In this work, we showed that *N, N’-*Squaramide 17 nanostructured into mimetic lipid membranes (MLS) presents an enhanced trypanocidal activity with decreased cytoxicity compared to free S. MLS was able to induce structural alterations in the parasites as fissures with a diameter of approximately 200 nm in epimastigote cytoplasm membrane. In summary, the high MLS activity against parasites demonstrates its target-specific toxicity, therefore suggesting that these nanostructures are promising for application in Chagas’ disease treatment. However, to confirm this MLS potential, further in vivo studies should be performed to compare MLS and S with respect to their trypanocidal activity, cytotoxicity and bioavailability.

## Figures and Tables

**Table 1:** Physical-chemical properties of the different nanostructures.

Nanostructure/Drugs	Hydrodynamic Diameter (nm) *	Polydispersity Index (PdI) *	Zeta Potential (mV) *
MLS	196.2 ± 11.0	0.418 ± 0.086	−61.43 ± 2.30
MLV	203.1 ± 8.5	0.428 ± 0.092	−12.93 ± 1.21

* The average of three independent experiments (mean ± standard deviation). Empty Mimetic Lipid Membranes (MLV) and Mimetic Lipid Membranes with *N, N’*-Squaramide 17 (MLS).

**Table 2:** Antiparasitic, cytotoxic activity and selectivity index of BZ, S, MLV, and MLS on *Trypanosoma cruzi* and on mammalian cells, respectively.

Nanostructure/Drugs	Epimastigote	Intracellular Amastigotes	VERO Cell	RAW 264.7 Macrophage	Epimastigote	Amastigotes in VERO Cell
	IC_50_ (μM) ^a^		CC_50_ (μM) ^b^		SI ^c^	
–	–	–	–	–	–	–
**Z**	15.81 ± 4.63	4.76 ± 4.45	284.44 ± 1.25	554.80 ± 4.90	17.99	59.76
**S**	13.12 ± 5.12	51.18 ± 4.91	736.21 ± 1.23	1654.377 ± 5.20	56.11	14.38
**MLV**	–	–	–	–	–	–
**MLS**	15.85 ± 4.82	24.92 ± 4.80	1199.50 ± 1.22	1973.97 ± 5.98	75.68	48.14

Benzonidazole (BZ), *N, N’-*Squaramide 17 (S), Empty Mimetic Lipid Membranes (MLV) and Membrane Lipid Membranes with *N, N’*-Squaramide 17 (MLS). Means of three independent experiments (mean ± standard deviation). ^a^ IC_50_ corresponds to the minimum concentration to inhibit 50% of epimastigote and amastigote forms. Cells were treated with 6.25 μM to 100 μM and incubated for 72 h. ^b^ CC_50_ in VERO cells and RAW 264.7 lineage macrophages. Cells were treated with 50 μM to 800 μM and incubated for 72 h. ^c^ Selectivity index (SI) represents = CC_50_ (VERO cell)/IC_50_ (extracellular and intracellular forms).

## Supplementary Materials

The following are available online at https://www.mdpi.com/1996-1944/13/23/5505/s1 Figure S1. Characteristics of nanostructures analyzed by atomic force microscopy (AFM). (A) Mimetic lipid membranes containing *N, N’*-Squaramide 17 (MLS), (B) Empty mimetic lipid membranes (MLV). Acquired in dynamic mode. Scanning area 5 µm × 5 µm. Figure S2. Calibration curve for *N, N’*-Squaramide 17 in different concentrations for percentage. Table S1. Entrapment efficiency of *N, N’*-Squaramide 17 was measured using a biophotometer by the absorbance of the drug at 320 nm. The experiments were performed in triplicate. Figure S3. Vero cells infected with *T. cruzi* (arrows). Cells were panoptic-stained. Figure S4. Morphological characteristics of nanostructures analyzed by transmission electron microscopy (TEM). (A) Mimetic lipid membranes containing *N, N’*-Squaramide 17 (MLS) and (B) Empty mimetic lipid membranes (MLV). Figure S5. Morphological alterations in *T. cruzi* after compound treatment analyzed by SEM. Epimastigotes treated with Benznidazole at 7.91 µM (BZ); *N, N’*-Squaramide 17 at 6.56 µM (S) and Mimetic lipid membranes containing *N, N’*-Squaramide 17 at 7.93 µM (MLS) for 72 h. (A) Control (without treatment); (B) BZ; (C) S; and (D) MLS.
