# Supplementary material for: Liposomes Composed by Membrane Lipid Extracts from Macrophage Cell Line as a Delivery of the Trypanocidal N,N’-Squaramide 17 towards Trypanosoma cruzi
Source: Materials (Basel). 2020 Dec 2;13(23):5505. doi: 10.3390/ma13235505 (PMC7730638; doi:10.3390/ma13235505)
Supplement: Supplementary file 1 [file materials-13-05505-s001.pdf]

materials

Supplementary Materials: Liposomes Composed by Membrane Lipid Extracts from Macrophage Cell Line as a Delivery of the Trypanocidal N, N′-Squaramide 17 towards *Trypanosoma cruzi*

Christian Rafael Quijia [1,2,3], Cínthia Caetano Bonatto [2], Luciano Paulino Silva [2], Milene Aparecida Andrade [3], Clenia Santos Azevedo [3], Camila Lasse Silva [3], Manel Vega [4], Jaime Martins de Santana [3], Izabela Marques Dourado Bastos [3,*] and Marcella Lemos Brettas Carneiro [1,*]

[1] Microscopy Laboratory, Department of Cell Biology, Institute of Biology, University of Brasília, UnB - Brasilia, Federal District, Brasília DF, 70910-900, Brazil; christianqui47@gmail.com.
[2] Laboratory of Nanobiotechnology, Embrapa Genetic Resources and Biotechnology, Parque Estação Biológica, PqEB, Av. W5 Norte (final) Caixa Postal 02372, Brasília DF, 70.770-917, Brazil; cinthiabonatto@gmail.com (C.C.B.), luciano.paulino@embrapa.br (L.P.S.)
[3] Pathogen-Host Interface Laboratory, Department of Cell Biology, Institute of Biology, University of Brasília, UnB - Brasilia, Federal District, Brasília DF, 70910-900, Brazil; andrademilene@unb.br (M.A.A.), clenia.azevedo@gmail.com (C.S.A.), camila.lasse@gmail.com (C.L.S.), jsantana@unb.br (J.M.d.S.).
[4] Department of Chemistry, University of the Balearic Islands, Palma on the island of Majorca, Carretera de Valldemossa, km 7.5, 07122 Palma, Illes Balears, Spain; manel.vega@uib.es.
* Correspondence: dourado@unb.br (I.M.D.B.), marbretas@gmail.com (M.L.B.C.); Tel +55 61 3107 3051

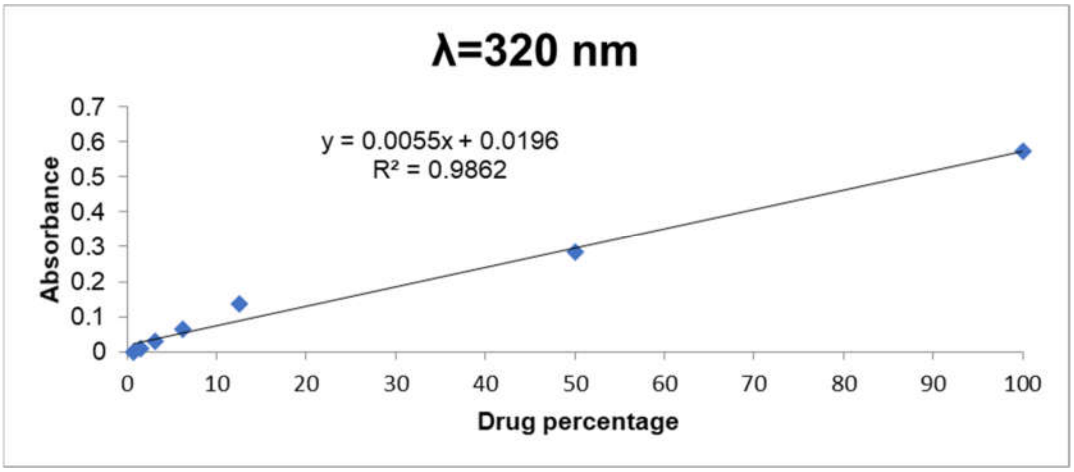

Figure S1. Calibration curve for *N, N′*-Squaramide 17 in different concentrations for percentage.

Table S1. The entrapment efficiency of *N, N′*-Squaramide 17 was measured using a biophotometer by the absorbance of the drug at 320 nm. The experiments were performed in triplicate.

**% Entrapment efficiency nanostructures- λ = 320 nm**	
1	75.71
2	72.07
3	72.35
Average	73.35
Standard deviation	2.05

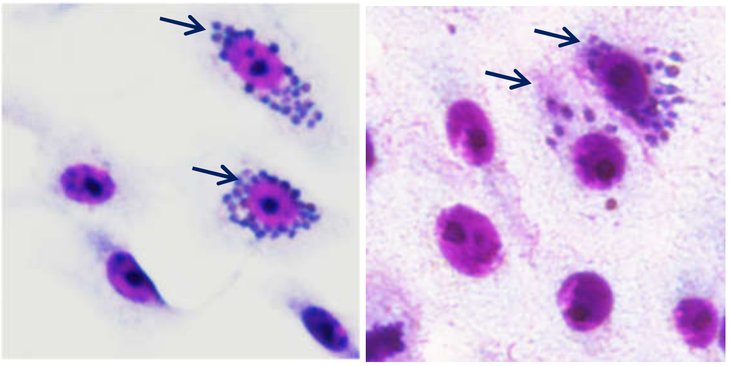

Figure S2. Vero cells infected with *T. cruzi* (arrows). Cells were panoptic-stained.

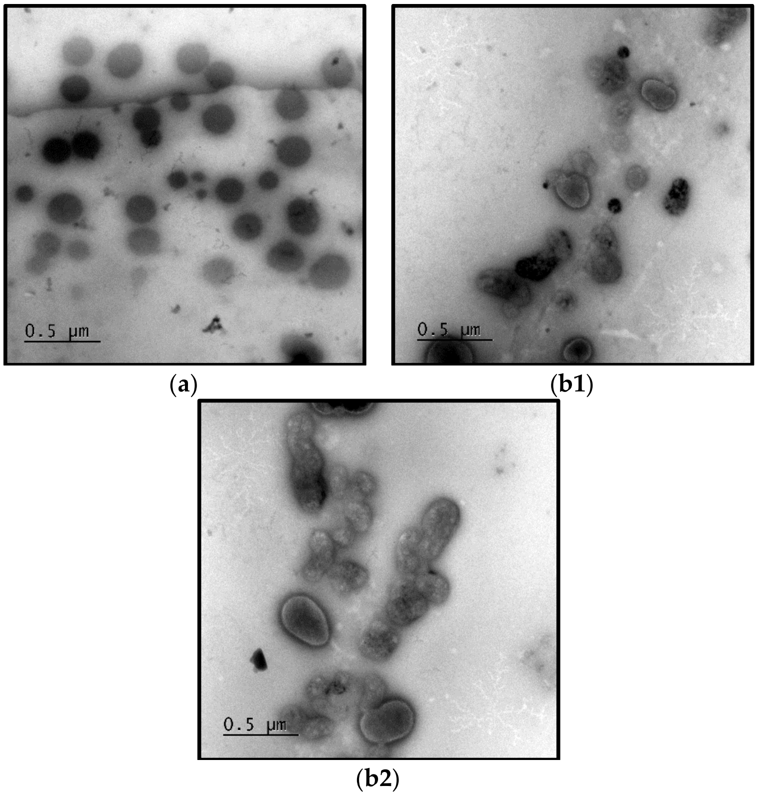

Figure S3. Morphological characteristics of nanostructures analyzed by transmission electron microscopy (TEM). (**a**) Mimetic lipid membranes containing *N*, *N′*-Squaramide 17 (MLS) and (**b1**, **b2**) Empty mimetic lipid membranes (MLV).

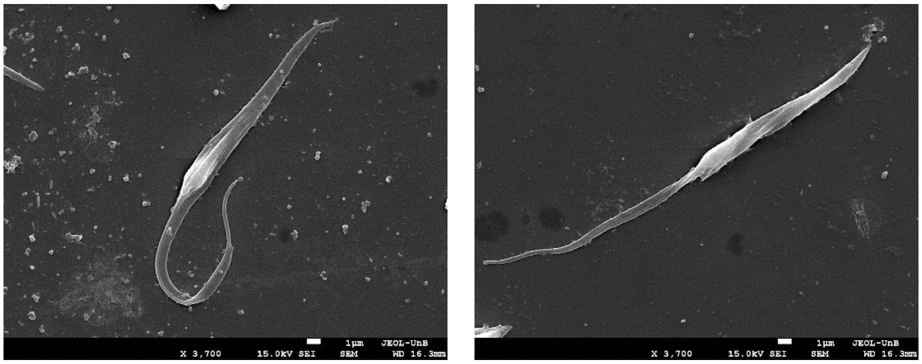

(**a1**) (**a2**)

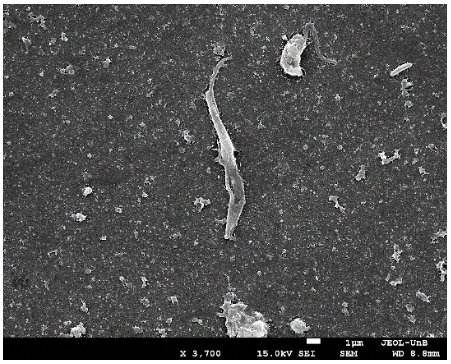

(**b**)

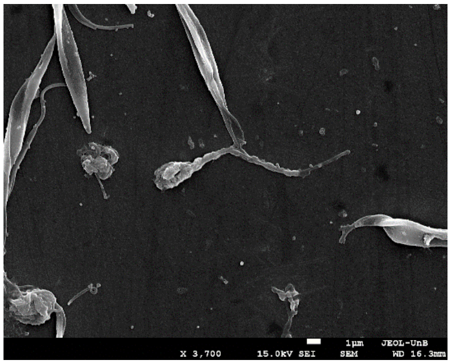

(**c1**)

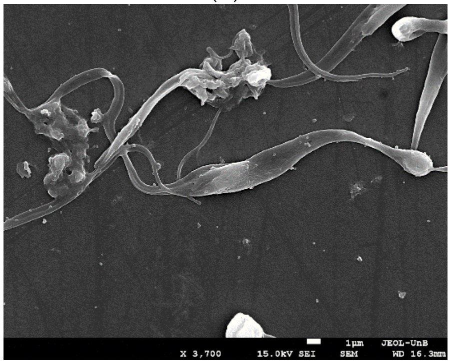

(**c2**)

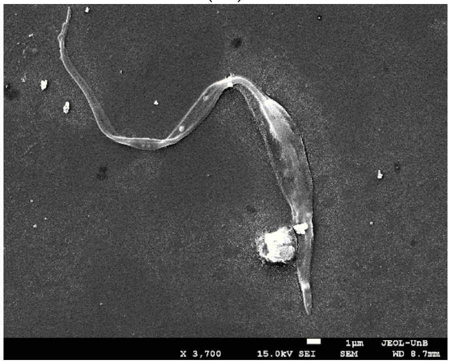

(**d1**)

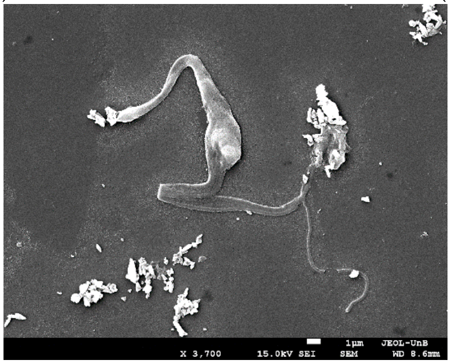

(**d2**)

Figure S4. Morphological alterations in T. cruzi after compound treatment analyzed by SEM. Epimastigotes treated with Benznidazole at 7.91 μM (BZ); *N, N′*-Squaramide 17 at 6.56 μM (S) and Mimetic lipid membranes containing *N, N′*-Squaramide 17 at 7.93 μM (MLS) for 72 h. (**a1**, **a2**) Control (without treatment); (**b**) BZ; (**c1**, **c2**) S; and (**d1**, **d2**) MLS.

Table S2. Hydrodynamic diameter and polydispersity index of the different nanostructures evaluated after one and ten days of their production under different pHs.

**Nanostructure (Conditions)**	**Day 1***			**Day 10***		
	**Hydrodynamic Diameter (nm)**	**Polydisper Sity Index (PdI)**	**Zeta Potential (mV)**	**Hydrodynamic Diameter (nm)**	**Polydisper Sity Index (PdI)**	**Zeta Potential (mV)**
**MLS**	196.2 ± 11.0	0.418 ± 0.086	−61.43 ± 2.30	126.5 ± 0.7	0,292 ± 0.029	−58.0 ± 3.89
**MLV**	203.1 ± 8.5	0.428 ± 0.092	−12.93 ± 1.21	360.0 ± 87.2	0.424 ± 0.091	−14.4 ± 3.32

*The data refer to the means of three separate experiments (mean ± standard deviation) measured by DLS. Empty Mimetic Lipid Membranes (MLV) and Mimetic Membrane Membranes with *N*, *N′*-Squaramide 17 (MLS).

© 2020 by the authors. Licensee MDPI, Basel, Switzerland. This article is an open access article distributed under the terms and conditions of the Creative Commons Attribution (CC BY) license (http://creativecommons.org/licenses/by/4.0/).
